# Supplementary material for: Amphibian gut microbiota shifts differentially in community structure but converges on habitat-specific predicted functions
Source: Nat Commun. 2016 Dec 15;7:13699. doi: 10.1038/ncomms13699 (PMC5171763; doi:10.1038/ncomms13699)
Supplement: Supplementary Information — Supplementary Figures and Supplementary Tables. [file ncomms13699-s1.pdf]

Supplementary Figures

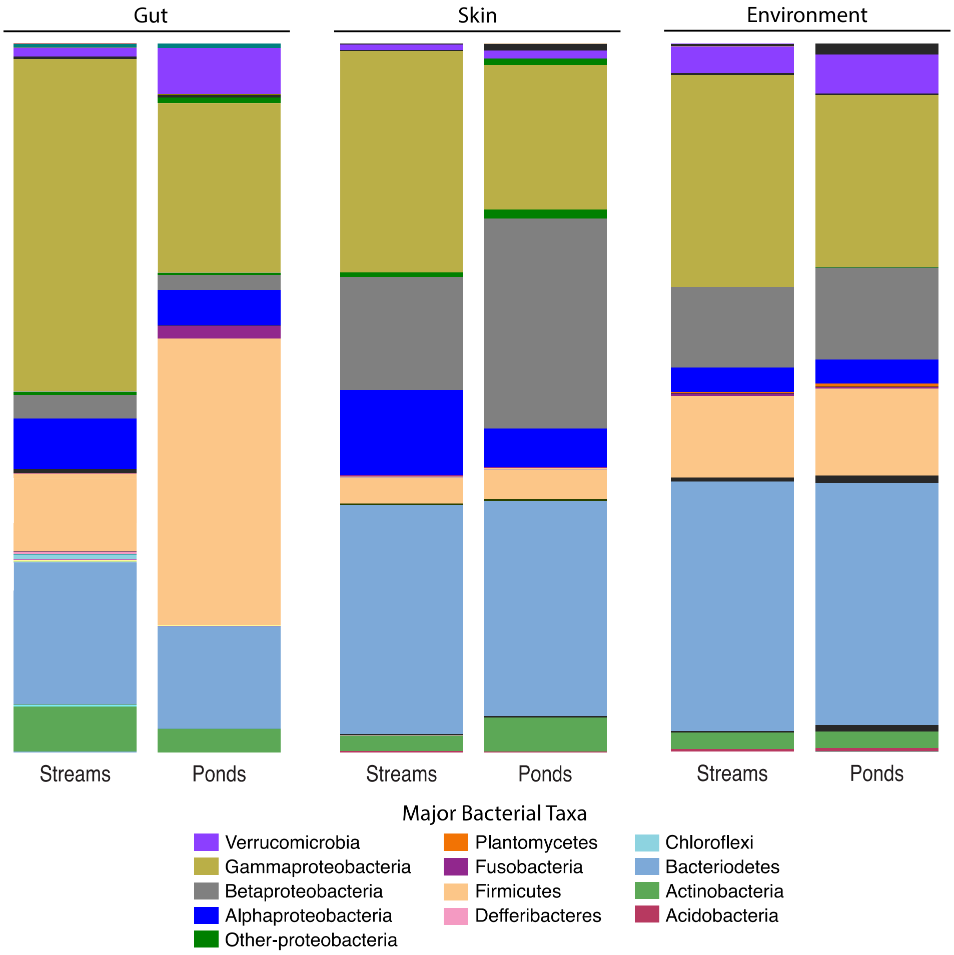

Supplementary Figure 1: Bacterial composition of gut and skin of salamander larvae and inhabited aquatic environments. Taxonomy is presented at the phylum level, with Proteobacteria divided out by class.

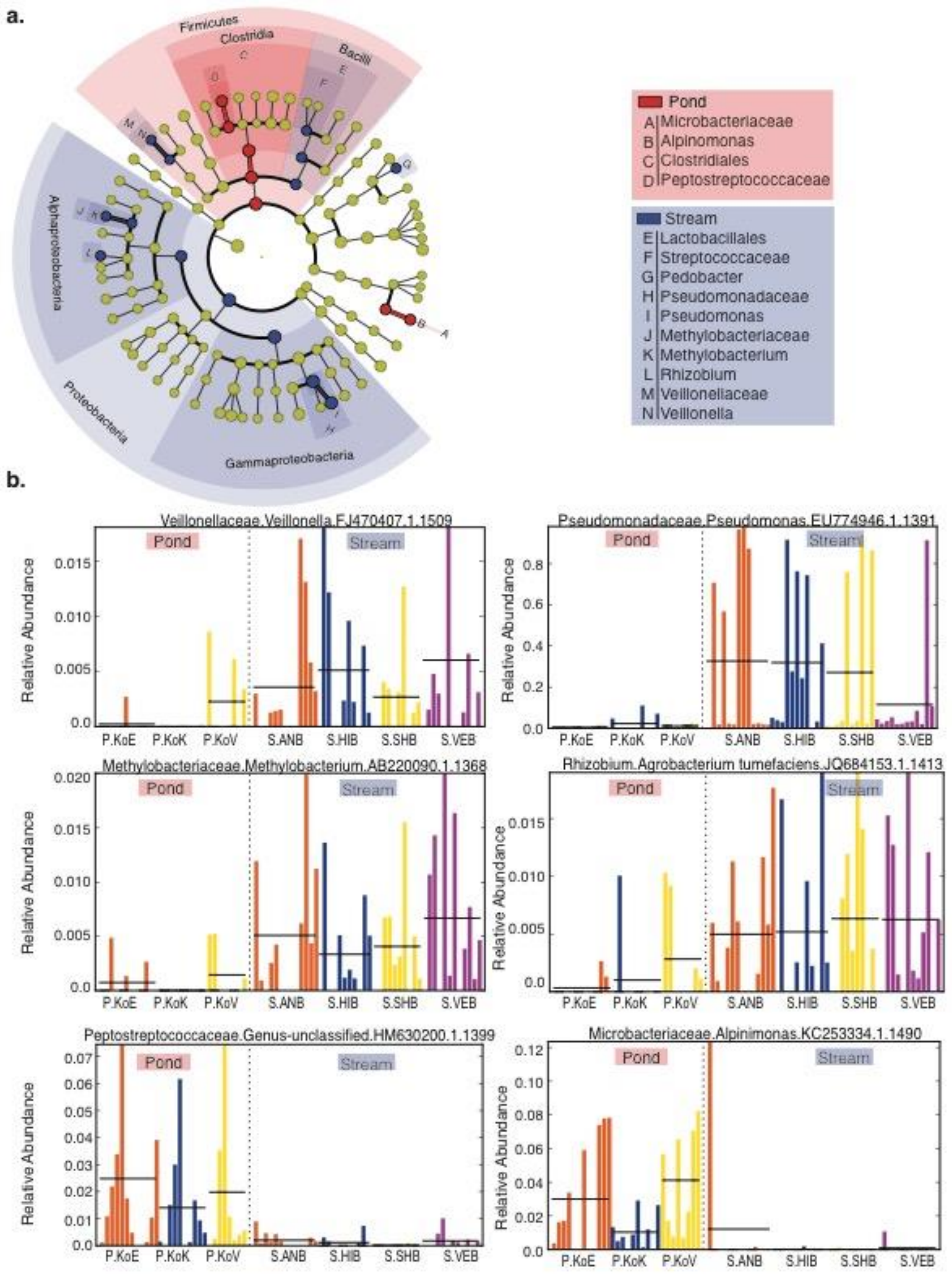

Supplementary Figure 2: LEfSe-detected taxa for pond versus stream larvae in field survey. (A) Cladograms of differential gut bacterial taxa detected by LEfSe. (B) Relative abundance of LEfSe–detected differential OTUs in pond and stream-type larvae gut bacterial communities, four OTUs found to be differentially abundant in stream larvae communities and two OTUs found to be differentially abundant in pond larvae communities.

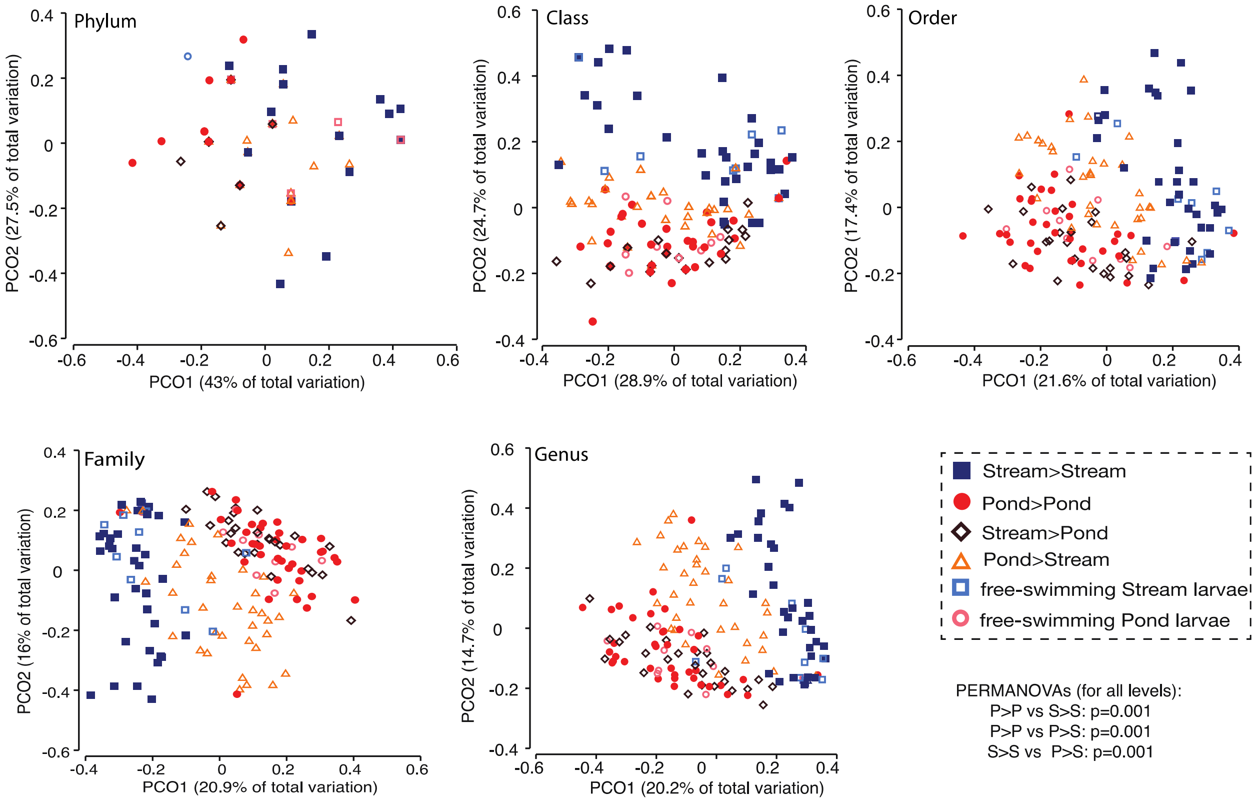

Supplementary Figure 3: Principal coordinate analysis of larvae gut bacterial communities at each taxonomic level. OTU tables were summarized at each level using QIIME and PCoA visualization was performed in Primer7. Importantly, the patterns observed are maintained at each level.

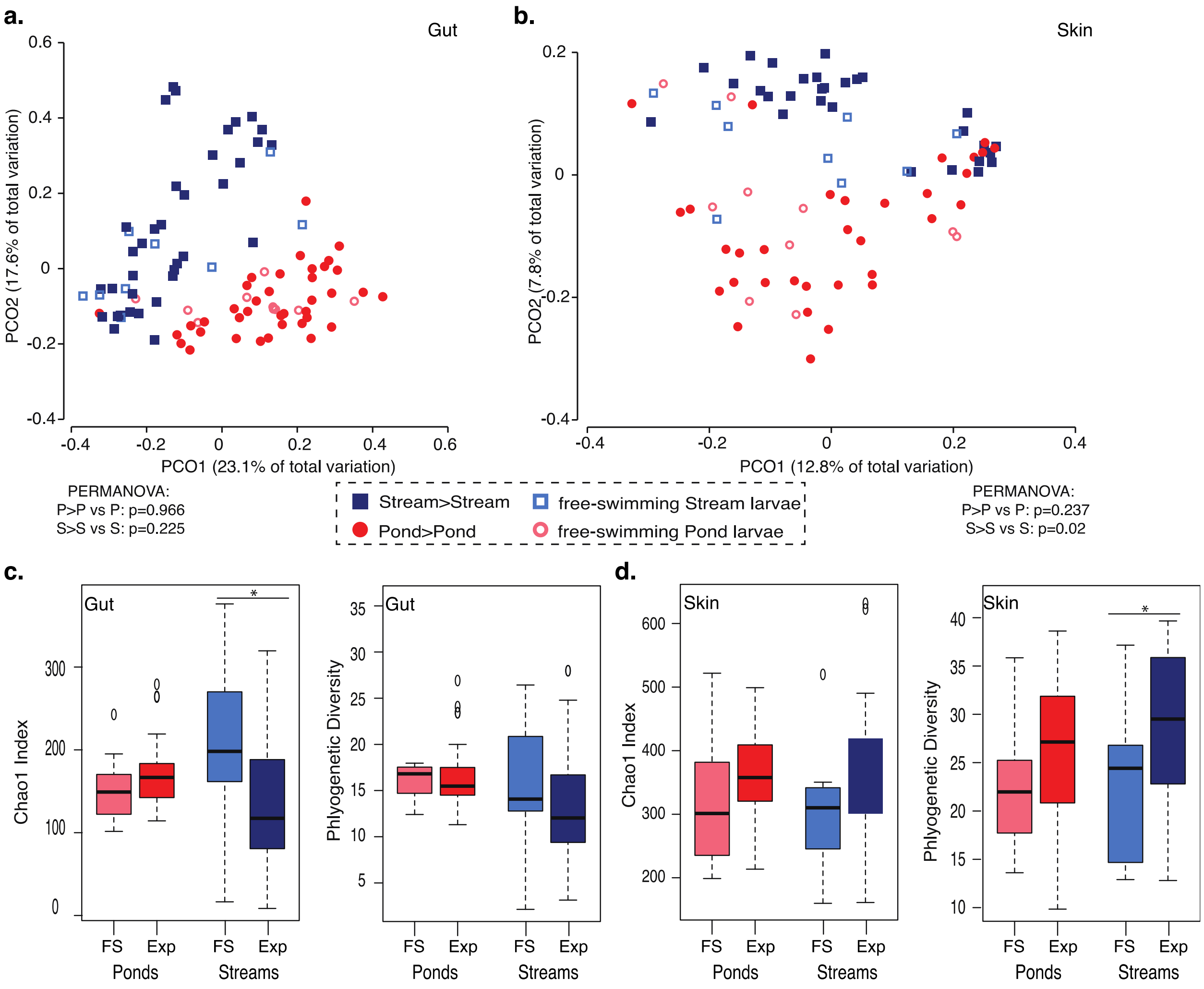

Supplementary Figure 4: Gut and skin bacterial communities of experimental larvae in comparison to free-swimming larvae. PCoA of unweighted Unifrac distances for gut bacterial communities (A) and skin bacterial communities (B). PERMANOVA results are provided below each PCo visualization. Alpha diversity using two metrics (Chao1 Index and Faith's phylogenetic diversity) for gut bacterial communities (C) and skin bacterial communities (D). asterisks denote instances of statistical differences as determined by Kruskal-Wallis tests.

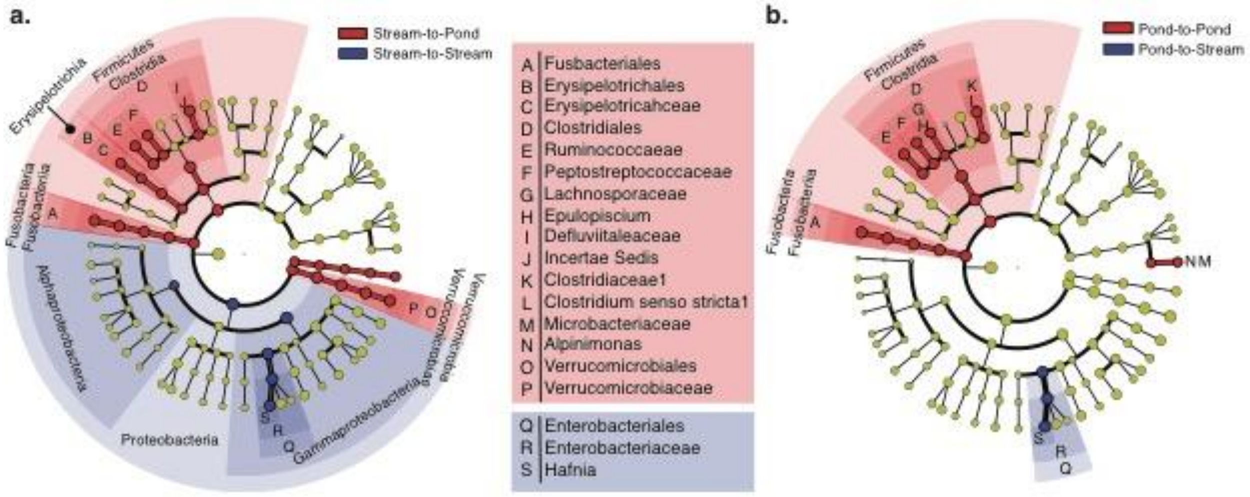

Supplementary Figure 5: Cladograms of differential gut bacterial taxa detected by LEfSe between each habitat-switched group and their respective origin habitat control group (A) differentially abundant taxa between the stream-to-pond group and the stream-to-stream group. (B) Differentially abundant taxa between the pond-to-stream group and the pond-to-pond group. Legend for both A and B is inset in the middle of the figure. Blue color identifies taxa with greater relative abundance in streams destination habitats and red color identifies taxa with great relative abundance in ponds destination habitats.

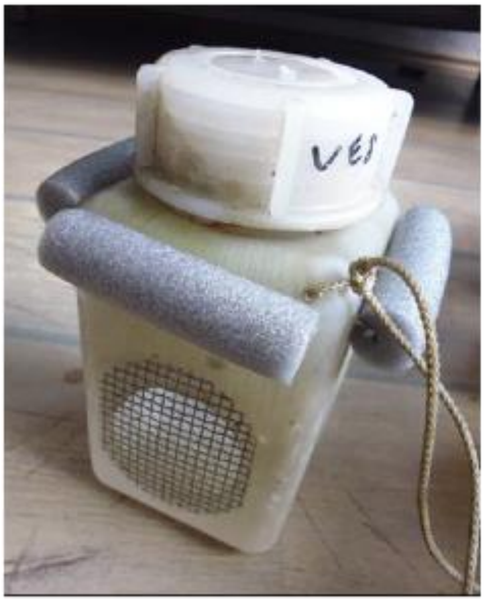

Supplementary Figure 6: Larvae housing enclosure from conducted reciprocal transfer experiment. Photo taken by D. Goedbloed.

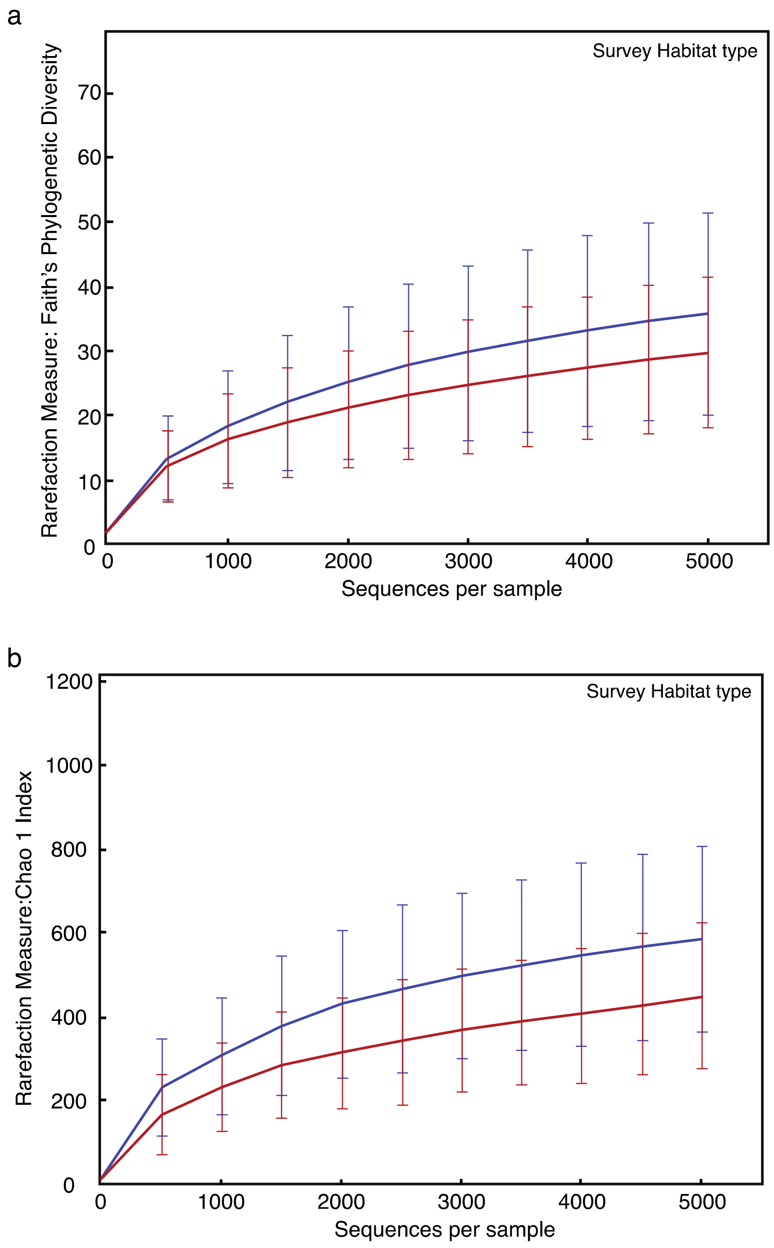

Supplementary Figure 7: Alpha rarefaction plots for Field Survey samples. (A) alpha rarefaction curves based on Faith's Phylogenetic Diverity (PD_whole_tree) for larvae gut bacterial communities from both habitat types, (B) alpha rarefaction curves based on chao1 diversity for larvae gut bacterial communities from both habitat types.

Supplementary Tables

Supplementary Table 1: Differential functional features of metabolism-associated gene pathways from pond and stream larvae gut bacterial communities.

Pond Differential Functional Groups (KEGG-level 3)	LDA Score	Stream Differential Functional Group (KEGG-level 3)	LDA Score
Nucleotide metabolism	2.10	Fluorobenzoate degradation	2.20
Xylene degradation	2.04	Atrazine degradation	2.10
Biosynthesis of ansamycins	2.10	Chlorocyclohexane and chlorobenzene degradation	2.32
Dalanine metabolism	2.17	Arachidonic acid metabolism	2.00
Novobiocin biosynthesis	2.22	Bisphenol degradation	2.24
Ascorbate and aldarate metabolism	2.09	Biosynthesis and biodegradation of secondary metabolites	2.35
Amino acid metabolism	2.31	Styrene degradation	2.24
C5Branched dibasic acid metabolism	2.14	Polycyclic aromatic hydrocarbon degradation	2.13
Photosynthesis	2.19	Synthesis and degradation of ketone bodies	2.27
Drug metabolism other enzymes	2.45	Lipid metabolism	2.35
Photosynthesis proteins	2.22	Metabolism of xenobiotics by cytochrome (P450)	2.51
Prenyltransferases	2.41	Drug metabolism cytochrome (P450)	2.58
Thiamine metabolism	2.48	Inositol phosphate metabolism	2.27
Nicotinate and nicotinamide metabolism	2.20	Caprolactam degradation	2.60
Galactose metabolism	2.88	Metabolism of cofactors and vitamins	2.32
Pentose and glucuronate interconversions	2.62	Biosynthesis of unsaturated fatty acids	2.53
Carbon fixation in photosynthetic organisms	2.59	Geraniol degradation	2.85
Pantothenate and CoA biosynthesis	2.43	Chloroalkane and chloroalkene degradation	2.01
Valine leucine and isoleucine biosynthesis	2.38	Lipopolysaccharide biosynthesis	2.60
Lysine biosynthesis	2.42	Glycosyltransferases	2.42
Peptidoglycan biosynthesis	2.52	Sulfur metabolism	2.04
Phenylalanine, tyrosine, tryptophan biosynthesis	2.41	Limonene and pinene degradation	2.60
Phosphotransferase system (PTS)	3.32	Phenylalanine metabolism	2.49
Starch and sucrose metabolism	2.94	Aminobenzoate degradation	2.69
Fructose and mannose metabolism	3.07	Protein kinases	2.58
Pentose phosphate pathway	2.82	Inorganic ion transport and metabolism	2.04
Aminoacyl tRNA biosynthesis	2.76	Glutathione metabolism	2.64
Alanine aspartate and glutamate metabolism	2.30	Beta-alanine metabolism	2.56
Cysteine and methionine metabolism	2.78	Lysine degradation	2.73
Methane metabolism	2.50	Lipopolysaccharide biosynthesis proteins	2.87
Pyruvate metabolism	2.36	Tyrosine metabolism	2.27
Amino sugar and nucleotide sugar metabolism	3.03	Fatty acid biosynthesis	2.48
Glycolysis & Gluconeogenesis	2.98	Benzoate degradation	2.77
Amino acid related enzymes	2.66	Tryptophan metabolism	2.87
Pyrimidine metabolism	3.02	Fatty acid metabolism	2.77
Purine metabolism	2.89	Lipid biosynthesis proteins	2.74
		Glyoxylate and dicarboxylate metabolism	2.75
		Valine leucine and isoleucine degradation	3.04
		Nitrogen metabolism	2.69
		Propanoate metabolism	2.79
		Energy metabolism	2.42
		Glycine serine and threonine metabolism	2.69
		Arginine and proline metabolism	2.68

Functional category IDs (KEGG Ortholog level 3) and Least Discriminate Analysis scores from LEfSe analysis are provided.

Supplementary Table 2: LEfSe-detected taxa from comparisons between the stream to pond group and the origin habitat control group**.**

LEfSe-detected Taxa	Experimental Group	LDA Score
**LEfSe comparison of stream-to-stream and stream-to-pond larvae**		
Firmicutes	S>P	5.134
Firmicutes.Clostridia	S>P	5.092
Firmicutes.Clostridia.Clostridiales	S>P	5.092
Clostridia.Clostridiales.Defluviitaleaceae	S>P	4.972
Clostridiales.Defluviitaleaceae.IncertaeSedis	S>P	5.006
Defluviitaleaceae.IncertaeSedis.JQ608165_1_1420	S>P	4.871
Clostridia.Clostridiales.Peptostreptococcaceae	S>P	4.212
Clostridia.Clostridiales.Ruminococcaceae	S>P	4.873
Clostridiales.Ruminococcaceae.New_ReferenceOTU606	S>P	4.889
Firmicutes.Erysipelotrichia	S>P	4.266
Firmicutes.Erysipelotrichia.Erysipelotrichales	S>P	4.346
Erysipelotrichia.Erysipelotrichales.Erysipelotrichaceae	S>P	4.280
Erysipelotrichales.Erysipelotrichaceae.New_ReferenceOTU512	S>P	4.307
Fusobacteria	S>P	4.940
Fusobacteria.Fusobacteriia	S>P	4.943
Fusobacteria.Fusobacteriia.Fusobacteriales	S>P	4.938
Fusobacteriia.Fusobacteriales.DQ814829_1_1434	S>P	4.953
Verrucomicrobia	S>P	5.140
Verrucomicrobia.Verrucomicrobiae	S>P	5.140
Verrucomicrobia.Verrucomicrobiae.Verrucomicrobiales	S>P	5.140
Verrucomicrobiae.Verrucomicrobiales.Verrucomicrobiaceae	S>P	5.140
Verrucomicrobiales.Verrucomicrobiaceae.New_ReferenceOTU197	S>P	5.147
Proteobacteria	S>S	5.501
Proteobacteria.Alphaproteobacteria	S>S	3.622
Proteobacteria.Gammaproteobacteria	S>S	5.490
Proteobacteria.Gammaproteobacteria.Enterobacteriales	S>S	5.239
Gammaproteobacteria.Enterobacteriales.Enterobacteriaceae	S>S	5.239
Enterobacteriales.Enterobacteriaceae.Hafnia	S>S	5.053
Enterobacteriaceae.Hafnia.AMQL01000001_12248_13793	S>S	5.053
Pseudomonadales.Pseudomonadaceae.Pseudomonas.EU774946_1_1391	S>S	4.804

Experimental group denotes in which group the taxa was more abundant.

Supplementary Table 3: LEfSe-detected taxa from comparisons between pond to stream transplant group and the origin habitat control group.

LEfSe-detected Taxa	Experimental Group	LDA Score
**LEfSe comparison of pond-to-pond and pond-to-stream larvae**		
Actinobacteria.Micrococcales.Microbacteriaceae	P>P	4.159
Micrococcales.Microbacteriaceae.Alpinimonas	P>P	4.310
Microbacteriaceae.Alpinimonas.KC253334_1_1490	P>P	4.279
Firmicutes	P>P	5.056
Firmicutes.Clostridia	P>P	5.087
Firmicutes.Clostridia.Clostridiales	P>P	5.087
Clostridia.Clostridiales.Clostridiaceae1	P>P	4.449
Clostridiales.Clostridiaceae1.Clostridiumsensustricto1	P>P	4.451
Clostridiaceae1.Clostridiumsensustricto1.EF590059_1_1364	P>P	4.453
Clostridia.Clostridiales.Lachnospiraceae	P>P	4.009
Clostridiales.Lachnospiraceae.Epulopiscium	P>P	3.948
Lachnospiraceae.Epulopiscium.New_ReferenceOTU440	P>P	4.018
Clostridia.Clostridiales.Peptostreptococcaceae	P>P	4.473
Clostridiales.Peptostreptococcaceae.DQ129557_1_1460	P>P	4.243
Clostridiales.Peptostreptococcaceae.HM630200_1_1399	P>P	4.120
Clostridia.Clostridiales.Ruminococcaceae	P>P	4.448
Clostridiales.Ruminococcaceae.New_ReferenceOTU606	P>P	4.447
Fusobacteria	P>P	4.849
Fusobacteria.Fusobacteriia	P>P	4.856
Fusobacteria.Fusobacteriia.Fusobacteriales	P>P	4.840
Fusobacteriia.Fusobacteriales.DQ814829_1_1434	P>P	4.846
Proteobacteria.Gammaproteobactieria.Enterobacteriales	P>S	4.748
Gammaproteobacteria.Enterobacteriales.Enterobacteriaceae	P>S	4.748
Enterobacteriales.Enterobacteriaceae.Hafnia	P>S	4.706
Enterobacteriaceae.Hafnia.AMQL01000001_12248_13793	P>S	4.705

Experimental group denotes in which group the taxa was more abundant.
